# Heterogeneity in Lateral Distribution of Polycations at the Surface of Lipid Membrane: From the Experimental Data to the Theoretical Model

**DOI:** 10.3390/ma14216623

**Published:** 2021-11-03

**Authors:** Rodion J. Molotkovsky, Timur R. Galimzyanov, Yury A. Ermakov

**Affiliations:** Laboratory of Bioelectrochemistry, A.N. Frumkin Institute of Physical Chemistry and Electrochemistry, Russian Academy of Sciences, 31/4 Leninskiy Prospekt, 119071 Moscow, Russia; gal_timur@yahoo.com

**Keywords:** lipid membrane, liposome, lipid monolayer, polycation adsorption, polylysine, electrokinetic potential, molecular dynamics

## Abstract

Natural and synthetic polycations of different kinds attract substantial attention due to an increasing number of their applications in the biomedical industry and in pharmacology. The key characteristic determining the effectiveness of the majority of these applications is the number of macromolecules adsorbed on the surface of biological cells or their lipid models. Their study is complicated by a possible heterogeneity of polymer layer adsorbed on the membrane. Experimental methods reflecting the structure of the layer include the electrokinetic measurements in liposome suspension and the boundary potential of planar bilayer lipid membranes (BLM) and lipid monolayers with a mixed composition of lipids and the ionic media. In the review, we systematically analyze the methods of experimental registration and theoretical description of the laterally heterogeneous structures in the polymer layer published in the literature and in our previous studies. In particular, we consider a model based on classical theory of the electrical double layer, used to analyze the available data of the electrokinetic measurements in liposome suspension with polylysines of varying molecular mass. This model suggests a few parameters related to the heterogeneity of the polymer layer and allows determining the conditions for its appearance at the membrane surface. A further development of this theoretical approach is discussed.

## 1. Introduction

Natural and synthetic charged macromolecules are widely used in industrial and biomedical applications [1,2,3,4,5,6]. Positively charged polymers (polypeptides and polycations of a different kind) are preferred in the latter case, since they interact with high effectiveness with the negatively charged biological membranes, containing a large amount of anionic lipids or negatively charged glycocalyx [7]. A number of studies provide evidence of antimicrobial mechanism of cationic biopolymers [8,9]. For instance, synthetic linear polymers, such as polylysine (PLL) and polyethyleneimine (PEI), are used in some targeted gene and drug delivery systems in complex with DNA and other molecules [10,11,12]. PEI and PEI-based nanoparticles (nanoPEI) demonstrate antimicrobial and antibiofilm activity [13,14], as well as polyvinylamines combined with pharmacologically active agents [15,16]. Some polycations, such as polyallylamine hydrochloride (PAH) or chitosan, are used as a cushion in the complex models of biomembranes [17,18,19]. The key factor that determines the effectiveness of the majority of biomedical and biotechnological applications of charged polymers is the number of macromolecules adsorbed at the surface of cell membranes, forming a stable bond with ionized components of their lipid matrix. Therefore, artificial lipid models of biomembranes composed of charged and electrically neutral components are of particular interest in the biophysical studies of these systems [20]. The electrostatic phenomena are commonly accepted as the most important factor accompanying the interaction of polycations with the charged surface of the membrane [21,22]. This raises the question about the role of polymer structure, size, and ionization in the interaction with the membrane. This question is intensively studied on the biomembrane models represented by liposomes, bilayer lipid membranes (BLM), and lipid monolayers [23,24,25]. These factors were discussed for antibacterial PEI-PMOX copolymers, and also for model lipid bilayers [26].

Interaction of large polycation molecules with the membrane surface qualitatively differs from that of many inorganic cations, as it follows from experimental data and theoretical analysis for monovalent [27] and most multivalent cations [28]. This discrepancy primarily owes to the presence of a spatially extended structure of charged macromolecules, which cannot be considered as concentrated as point-like charges. This factor can lead to the formation of a laterally inhomogeneous polymer layer [29], expressing as incomplete patch-like polymer coverage of the surface. For the sake of simplicity, we refer to a similar coating as the adsorption layer. Here and below, the term “lateral” is related to the distribution along the membrane surface as opposed to normal direction. Its heterogeneity should become more prominent when the membrane is composed of the lipid mixture. Because of the liquidity of the membrane, charged components of the mixture can redistribute in the presence of the adsorbed polyelectrolyte and thereby facilitate heterogeneity of the polymer layer [30,31]. A detailed study of related phenomena is complicated because it is not easy to combine the general physical characteristics of the adsorption layer with the details of the individual structure of the macromolecule at the membrane surface. In the review, we systematically analyze the methods of experimental registration and theoretical description of the laterally heterogeneous structures in the polymer layer published in the literature and in our previous studies.

There are three different approaches concerning the adsorption of polycations on the charged surfaces: experimental methods [32,33,34], those based on the general theoretical physical models [35,36,37], and various techniques of the numerical simulations [38,39,40,41,42]. These approaches explore fundamentally different characteristics of adsorption: the averaged characteristics of the adsorption layer measured in the experiments; simplified physical parameters of the system used by the theoretical models, and the quantitative description of simulated systems at the molecular level obtained from numerical simulations. These approaches are complementary and therefore provide a unique opportunity to obtain an integral picture of the possible inhomogeneities at the membrane surface revealed, or generated, by polycation adsorption. Electrostatics is well known to play a decisive role in the interaction of polycations with a charged surface. The physical mechanisms of this interaction are analyzed in detail by many authors. See, for example, comprehensive reviews, referenced to many original studies in this field [31,43]. We realize that our analysis of literature data does not exhaust the variety of effects caused by polymer interaction with cell membranes. We focus our attention on inhomogeneous structures on the membrane surface in the presence of a polymer layer.

The review is structured as follows: firstly, we discuss the physical mechanisms involved in the adsorption of polyelectrolytes closely related to the electrostatic phenomena at the membrane boundaries. In this section, we briefly describe some bioelectrochemical methods that enable separating polycation effects on the different components of the electric potential near the membrane surface and inside of its polar region. Next, the similarity and fundamental difference between polycations and multivalent inorganic ions are analyzed in the context of known experimental data and theoretical description of their adsorption at the lipid membranes. Then, in a concise form, we characterize the effect of polycations on the membrane surface; in particular, the possibility of segregation of negatively charged components of the membrane is described. Further, we turn to the conformations of the adsorbed macromolecules and discuss data indicating their alterations on the membrane surface. In the final section, we present parameters of the model developed recently to describe electrokinetic data on polylysine adsorption on the surface of liposomes. A simple theoretical approach allows us to form a view about the lateral heterogeneity of the polylysine layer at the membrane surface, to suggest parameters characterizing this heterogeneity and to predict the favorable conditions for their experimental control.

## 2. Electrostatic Contribution of Polymers’ Interaction with Phospholipids

Adsorption of polycations and charged peptides on the surface of lipid membranes is a subject of various methods and their quantitative analysis is discussed in detail in a review [44]. A rich experimental material was obtained by the methods of fluorescence spectroscopy [45,46,47], atomic force microscopy [48,49,50], ellipsometry [43,51], by the technique of Langmuir monolayers [52,53], and by registration of the electrokinetic mobility in colloid suspensions [54,55,56]. In most cases, these methods detect the averaged characteristics of the polymer adsorption layer such as the thickness or potential drop on the membrane/solution boundary that cannot always be quantitatively related to the amount of adsorbed substance. Some conclusions about the lateral distribution of polymer on the surface follow from data of atomic force microscopy (AFM) methods, which allow estimating the fraction of the surface occupied by the polymer.

Below, we present the most interesting results related to the effect of polycation molecules adsorption on different components of the electric field at the membrane boundary obtained by electrokinetic and alternative methods. A combination of experiments and numerical molecular simulations of lipid–polymer systems provide information sufficient to develop physical models of these systems. Methods of molecular dynamics (MD) in full atomic resolution are commonly used for numerical simulations of the systems of limited size. They visualize the membrane and distribution of polymers, providing information on the hydration state of lipids and the difference between the conformation of macromolecules in the bulk and at the charged surfaces [40,57,58]. So-called “scaling theory” is often used to analyze and summarize the entire data collection of experiments and numerical simulations [59,60,61,62]. This approach provides a realistic estimation of polymer layer thickness for different modes of its adsorption, depending on the external parameters of the system and the ionic strength of the solution. However, the layer formation itself is not considered within the framework of this approach, and the corresponding adsorption isotherms are not proposed in cited works. There are a few examples in the literature where proper isotherms have been proposed, taking into account the electrostatic nature of charged polymer interaction with the surface [32,63].

As follows from the majority of experimental data, adsorption of polycations on the membranes composed from neutral or zwitterionic lipids practically does not occur. It was shown by electrokinetic measurements in liposome suspension in the presence of short peptides with several positively charged subunits [32], by nonlinear optical technique of second harmonic generation (SHG) [64], and from the analysis of differential scanning calorimetry and Fourier transform infrared spectrometer (FTIR) data in the case of polylysines of different states of polymerization [65]. However, there is an opposite point of view on this matter. Adsorption of some natural polycations (chitosans) has been observed on membranes of zwitterionic lipids (dioleoyl-phosphocholine, DOPC) [66,67]. This lipid remains uncharged over a wide pH range when exposed to the solution with high ionic strength, relevant to biological systems [68,69]. Weak negative charge of DOPC is observed at smaller ionic strength [70,71]. The origin of this charge is disputed, but typically hydroxide ion affinity is used as an explanation [70,72]. In works [66,67], authors provide data on a change in DOPC liposomes charge with pH, which becomes significantly negative (−30 mV of ζ-potential) at high pH values. It is not surprising that positively charged chitosan molecules can adsorb at such a surface. We may remark that in this specific case, a significant contribution of the electrostatic factor to their interaction was experimentally proved. The possible effect of pH on the adsorption of polycations was the subject of theoretical analysis in the work [73]. According to this analysis, the proton concentration near the negatively charged surface is increased, which reflects in significantly lowered pH value in comparison to the bulk solution. This in turn increases the degree of protonation and the positive charge of the polymer, which enhances their electrostatic interaction with the surface. If pH in the bulk corresponds to the isoelectric point of the polymer, then a decrease in pH nearby the surface can lead to the inversion of the polycationic charge, and even to the attraction of the polymer, to a positively charged surface. This effect can be additionally enhanced by the phenomenon of an entropic trap which keeps protons near the surface and ensures their channeling along the membrane interface, even in the absence of potent acceptors [74]. This factor should be taken into account in the development of the theoretical models and adsorption isotherm. Nonelectrostatic hydrophobic interactions can contribute to the adsorption of polymers to the lipid membranes in some cases, as mentioned below. In the case of strongly charged polymers such as polyethylenimine (PEI) and poly-L-lysine (PLL), electrostatic contribution to their interaction with the charged surface generally prevails over the hydrophobic one.

The details of the electrostatic interaction between polycations and phospholipids are well illustrated by the molecular dynamics simulations of these systems. According to the results of [75], positively charged molecules of poly ([3-(methacryloyl amino) propyl] trimethylammonium chloride (PMAPTAC) interact predominantly with anionic phosphatidylserine molecules and practically do not interact with zwitterionic palmitoyl-oleoyl-phosphocholine (POPC) molecules. Electrostatic interactions also control the adsorption of linear polymers of another type, polyethylenimine (PEI) and poly-L-lysine (PLL) [40]. The nature of these interactions is largely determined by the degree of polymer ionization, which is almost equal to unity in the case of PLL, and the presence of relatively long side groups. These groups can act as a steric factor, preventing the tight contact of molecules with the membrane. This effect was most clearly demonstrated in the case of PLL [40]. The same result is described in the work [76] for Lys8 and Arg8 oligopeptides. Polyarginine molecules turn out to be predominantly inserted into the membrane, while polylysine is partially exposed to the solution. Due to the large Born radius of arginine, its hydrophobic side groups in the polymer chain can insert into the region of lipid hydrocarbon tails, and at high surface density may create transmembrane pores [77]. On the other hand, lysine molecules demonstrate another type of behavior. Judging by the data of Raman spectroscopy, short polylysine molecules destabilize the membrane, while long ones can lead to the opposite, providing a stabilizing effect [78]. During adsorption, protonated groups of polypeptides form the hydrogen bonds with phosphate groups of anionic lipids, thus enhancing interaction with the membrane [40,57,79,80]. Alterations in the hydrogen bonds network in the polar region of the membrane were found even during the adsorption of individual lysine molecules (Lys_1_) [81]. During this process, water bound on phosphate groups is displaced, which contributes to the dipole potential recorded in the experiment. Quantitative analysis of MD simulations of this system indicates that in the absence of anionic lipids in the membrane, adsorption of model polymer molecule is driven by hydrophobic interaction of its side chains with hydrocarbon lipid tails [77]. The presence of such side chains leads to deeper insertion of polymer chains into the membrane and facilitates pore formation.

## 3. Inorganic Cations and Polycations in the Electric Fields at the Membrane Boundary

**Figure 1:**
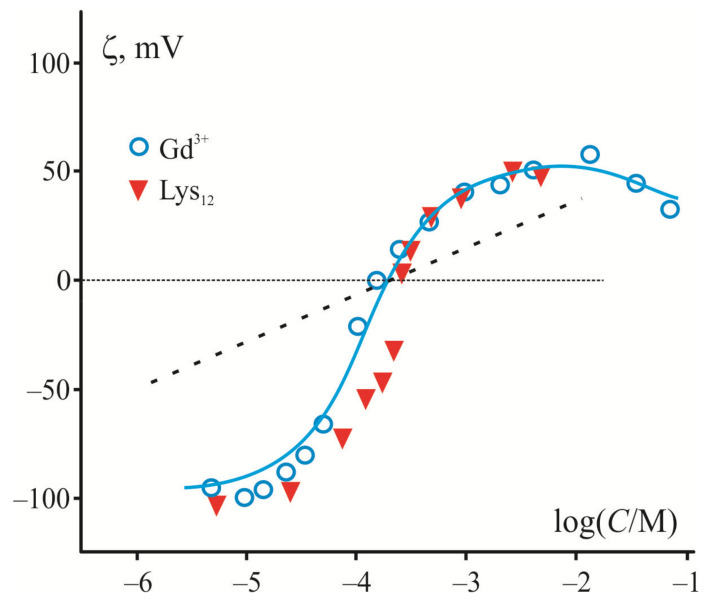
Variation of ζ–potential measured in the suspension of liposomes from anion phospholipids (PS) in the presence of multivalent cations and polylysines (data for Gd^3+^ and PL are taken from [82] and [83], respectively). Abscissa axis is presented in logarithm of moles of cation or subunits of polycation, correspondingly. The Gouy–Chapman theory predictions are presented as the maximal slope of curves for normal three-valence cations (dotted line). The solid line corresponds to the model adapted in [28] to the adsorption of Gd^3+^ with the extremely high affinity to phospholipids.

Since adsorption of large polycations and inorganic multivalent cations is largely determined by electrostatic interactions with the charged membrane surface, they demonstrate a similar shape of ζ-potential dependence on their contents measured in liposome suspension of a negatively charged lipid. The electrokinetic data in Figure 1 demonstrate this fact in the example of adsorption of trivalent cations of Gd^3+^ [82] and relatively short polylysine molecules (12–20 subunits) [83] on the surface of liposomes composed of phosphatidylserine (PS). In both cases, there is a sharp rise of the curve near the point of zero charge where the surface is neutral, and the electrostatic interactions are minimized. The magnitude of ζ-potential reaches its maximal value at the saturation level, which depends on the ionic strength and polymer length [84]. Note that in the case of multivalent cations, ζ-potential has a maximum and then decreases at high ion concentrations, whereas in the case of polylysines, this potential saturates. This discrepancy is caused by the fact that inorganic cations, but not polycations, participate in the screening inside the diffusion layer characterized by the Debye length. It is necessary to note here that electrokinetic measurements are related to the electric potential (ζ-potential) at the slipping plane at a certain distance from the charged surface [85]. Previously, adsorption of polylysine was measured at low ionic strength, when the ζ-potential becomes close to surface potential [84]. We accepted this condition to simplify the analysis and to evaluate the surface charge density according to the Smoluchowski relation and equations of the Gouy–Chapman model. This charge density was found independent of the ionic strength of the solution for each type of the polymers, while the surface potential was altered in full accordance with the prediction of the Gouy–Chapman model. A similar conclusion was drawn in a series of works by Duan et al. [86,87]. The authors applied the Monte Carlo method for the analysis of the efficiency of linear model polycation adsorption on the surface of membranes with different content of anionic lipids. They convincingly showed that increased ionic strength enhances the screening of the polymer charge and suppresses their adsorption, regardless of the degree of polymerization.

Generally, the application of the Gouy–Chapman model of the electric double layer in its straightforward simplified form to large polycations in [84] does not stand up to criticism. First of all, this rather simple model assumes the existence of a diffuse part of the electric double layer with the charge coinciding in absolute value with the surface charge of the opposite sign [85,88]. This layer contains mobile inorganic ions whose size is usually neglected. However, the latter assumption is invalid in the case of polymer molecules, which cannot be considered as point ions with a formal high “valence”. Then, a simple model application to electrokinetic measurements supposes that the slipping plane is located at some distance from the charged surface inside of the diffuse layer, where the screening effect of inorganic ions becomes the most significant and sensitive to the ionic strength of the solution. This fact is especially noticeable in the case of multivalent cations (see Figure 1) [28].

In any case, it is not possible to describe, within the framework of a simple model, the steep region of experimental curves observed in the electrokinetic measurements of adsorption of multivalent inorganic cations with an extremely high affinity to anionic phospholipids (Be^2+^, Gd^3+^, and other lanthanides) [28]. The maximal slope of these curves was calculated for point-like inorganic cations of different valence within the Gouy–Chapman model. It predicts a decrease of this slope by 60, 30, and 20 mV per decade of ion concentration for 1-, 2-, and 3-valence ions, respectively [28]. A dotted line in Figure 1 corresponds to the case of trivalent cations. Note that predictions of the model work well for the small organic molecules and cations, which do not noticeably affect the membrane structure. It was tested for a set of typical inorganic cations [28] and organic cations with a single positive charge, lysine [81] and chlorpromazine [89]. In contrast to these cases, a description of the electrokinetic data for multivalent cations of extremely high affinity to the membrane has to be modified to account for their depletion in the experimental cell during adsorption on the liposome suspension [90] (see theoretical curve in Figure 1). Since macromolecules cannot be considered as point-like charges, their presence in the diffuse part of the electric double layer has no direct effect on the Debye length, and the nature of their interaction with the surface fundamentally differs from that of inorganic cations. In particular, to analyze the participation of these molecules in the electric field distribution within the diffuse part of the electric double layer, one should solve the Poisson–Boltzmann equations, taking into account the charges of polymer subunits distributed in the polymer layer [91]. In this case, the question about the position of the slipping plane of colloidal particles and liposomes with a polymer layer at their surface remains discussible.

**Figure 2:**
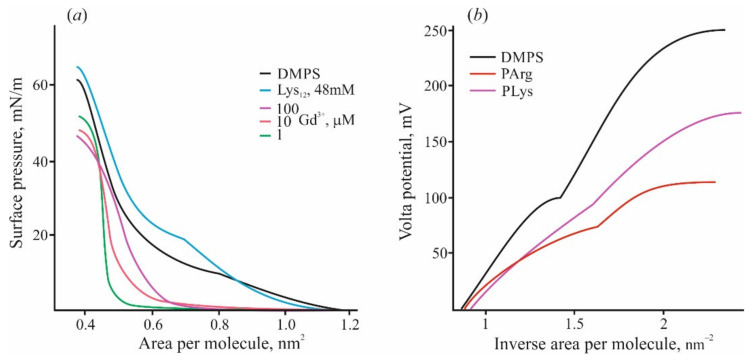
(**a**) Lateral pressure—lipid area diagrams measured by the Langmuir technique on monolayers of DMPS at the subphase of 10 mM KCl (black curve) and in the presence of Gd^3+^ of varied concentration or polylysine, shown by lines of different colors. (**b**) Volta potential of DMPS monolayers was measured at the subphase of 10 mM KCl (black line) and in the presence of polylysine and polyarginine (48 mM of bases) in the water subphase. Abscissa is presented in the scale of lipid density (1/nm^2^).

There are some other phenomena qualitatively distinguishing adsorption of polypeptides from the case of inorganic ions. Isothermal titration calorimetry (ITC) with DMPS liposomes titrated by high-affinity cations of Gd^3+^ reveals their participation in the lipid liquid–gel phase transition due to their ability to compact several neighboring lipid molecules into microclusters [90,92]. This fact is reflected in the compression diagrams of lipid monolayers (Figure 2a). In the presence of Gd^3+^ in water solution, the shape of these diagrams changes significantly, and the region with a liquid expanded state of lipid disappears [92]. On the contrary, the same diagram in the presence of polylysine preserves this region and shows the phase transition of the lipid to the condensed state. Adsorbed polymer only slightly raises the rigidity of the monolayer towards the compression and increases the pressure of passing into the condensed state. According to the data of the work [93], pentalysine lowers the pressure in the liquid phase and increases it in the gel phase, while the other peptides only decrease it with a decrease in the effect as the monolayer “solidifies”. Calorimetric measurements also show qualitative and quantitative differences in the heat effects induced by polycation and Gd^3+^ ions [92]. In both cases, the condensed gel phase appeared approximately at the point of zero charge. Polylysine adsorption on the DMPS liposomes in the liquid state demonstrated heat consumption significantly (about 40 times) lower compared with Gd^3+^ ions. When the measurements are carried out with lipids in the gel state, the heat release (exothermic effect) is 10 times weaker for monolayers with adsorbed polycations.

A significant part of the boundary potential falls within the polar region of phospholipids, which makes it inaccessible for electrokinetic measurements. In most cases, it is associated with the orientation of the dipole moments of water molecules and individual polar groups of lipids. It is natural to identify it with the dipole component of the boundary potential. In particular, its changes reflect the influence of multivalent inorganic ions, charged molecules, and polymers on the state of hydration and lateral packing of lipids [81,94,95]. To control these changes, we use the combination of electrokinetic data and two alternative methods, sensitive to a full potential drop at the membrane interface, i.e., between hydrophobic area of the lipid bilayer and the bulk of the electrolyte. The technique developed in our laboratory uses the intramembranous field compensation (IFC) to detect the difference of the boundary potentials across the planar BLM or at one membrane side if another is kept as the reference. A detailed description of this method is presented in our previous publications and reviews [28,96]. This technique allows registration of the boundary potential kinetics altered due to the adsorption of any substances at one membrane side. It can be used to test the reversibility of their adsorption by continuous perfusion of the experimental cell with a background solution. Particularly, this method demonstrates the complete reversibility of the studied inorganic cations and small organic molecules (lysine) adsorption [81,90]. On the contrary, polylysines are not removed from the membrane by prolonged perfusion because they are bound to the membrane surface almost irreversibly, though adsorbed without insertion into the membrane. This conclusion was confirmed by electrokinetic measurements of polymer adsorption in the liposome suspensions with varied lipid contents [97]. The small amount of lipid leads to a parallel displacement of the experimental curves towards a smaller amount of polymer required to reach the zero-charge point, while the limiting value of ζ-potential at saturation level remains unchanged. This means that the equilibrium in the suspension is strongly shifted towards polymer adsorption, which is extremely efficient and irreversibly covers the entire accessible surface of the liposomes in the suspension. The irreversibility of adsorption of polycations with a relatively low degree of polymerization (Lys_20_) also follows from its comparison with pentalysine (Lys_5_), as shown in MD simulations in our recent work [98]. Molecules of Lys_20_ in the simulation remained bound to the membrane during the entire simulation time, whereas pentalysine was found in a partially desorbed state.

**Figure 3:**
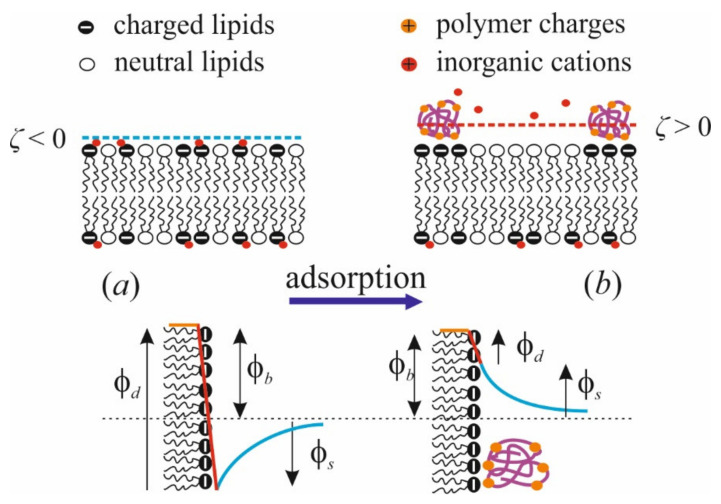
Schematic representation of the effects of polycation adsorption on the membrane structure (upper row) and components of the boundary potential of exposed membrane side (*φ_b_*): surface (*φ_s_*) and dipole (*φ_d_*) (lower row). (**a**): Initial state of the membrane; (**b**): membrane with the polycation layer.

Applying the method of IFC, we successfully showed the change of the boundary potential in response to polycation adsorption as complex two-stage kinetics [97]. At the initial rapid step of kinetics, these changes are positive, as would be expected from electrokinetic data. Then, there is a slow alteration in the opposite direction with a small magnitude equal for polymers of different lengths. Long-term perfusion of the cell does not reveal further changes in the boundary potential; therefore, we attributed the second step not to polymer desorption, but to some alteration in the polar region of the membrane. The sum of the initial potential jumps quantitatively coincides with the maximal variation of ζ-potential at similar conditions. The potential drop in the negative direction is quantitatively consistent with a similar effect measured with nonpolymerized lysine molecules [81]. We associate this drop with the effect of lysine on the hydration state of phosphate groups. This is illustrated schematically below in Figure 3. This conclusion is also confirmed by MD simulations [81]). On the contrary, multivalent cations Gd^3+^ [92] and Be^2+^ [99] show the dipole effect of the positive sign. It is explained as the reduction of the polar groups hydration, facilitating the lipid phase transition to the condensed state.

Since almost all main anionic phospholipids under normal conditions (pH about 7.0) have a single negative charge, the binding of two- and three-valence inorganic cations to the polar groups of these lipids not only compensates their charge, but even changes the sign of surface charge to the opposite and overcharges the surface. In the particular case of divalent cations, the surface charge becomes zero when half of the binding sites are occupied by cations [28,100]. The corresponding concentration is equal to the inverse association constant. The nature of the overcharging substantially differs in the case of polycation adsorption. During the adsorption of polycations, macromolecular chains tend to preserve as much configurational entropy as possible, leading to the formation of loops and tails which are not completely attached to the surface. This leads to the fact that there are more charged polycation subunits directly or indirectly attached to the surface than the number of binding sites of the surface.

Overcharge of the surface by polyelectrolytes is a fundamental physical phenomenon. It is used, for example, for the formation of multilayer films by layer-by-layer adsorption of polyanions and polycations, for which final complex structure and thickness can be controlled at the molecular level [101,102,103]. The same phenomenon largely determines the process of flocculation caused by the aggregation of identically charged particles [104], and the adsorption of proteins on identically charged surfaces. Detailed study of these effects can be found in [105,106,107,108], and the theoretical analysis, carried by mean-field methods can be found in [109]. It was shown that the overcharge phenomenon always occurs at low ionic strength. In this case, the electrostatic interaction acts at a greater distance, and the polymers in the solution experience strong attraction from any oppositely charged surface. Adsorption continues until the effective surface charge acquires the same sign as molecules of adsorbent. In the high ionic strength limit, the surface electric field is more screened, and charge inversion occurs when the short-range interaction between monomers and the surface is not too strong, and the surface charge is not too high.

The value of boundary potential under certain conditions coincides with the Volta potential measured by the Langmuir lipid monolayer technique. Recently, we carried out a series of measurements by compression of lipid monolayers on the water–air interface exposed to various substances [89]. The changes of Volta potential measured simultaneously with compression diagrams usually demonstrate a region with linear dependence on the lipid density at the water–air interface. The slope of this dependence can be used for the evaluation of the effective dipole moment [110], according to the Helmholtz relation. The slope of this region for DMPS monolayer and the corresponding value of the dipole moment noticeably increases in the process of the adsorption of multivalent cations [92,99], while the presence of polylysine significantly decreases this value (see Figure 2b). Previously, we studied the monolayer of DMPS in the liquid and condensed states by small-angle X-ray scattering and by numerical analysis of molecular dynamics simulations [111]. Water molecules associated with the polar groups of the phospholipid were found to be responsible for the changes in Volta potential and the value of the effective dipole moment. Note that arginine-based polycations in the same region cause deviation from the linearity of the curve (Figure 2b). We hypothesize that, in this case, the electrostatic interaction of the polycation with the surface is accompanied with the partial penetration of the polymer into the monolayer polar region, which disappears upon compression. A similar effect was observed for adsorption of small positively charged molecules of chlorpromazine, which inserted into the lipid monolayer upon adsorption [89]. All results presented in this section indicate a fundamentally different effect on the structure of membranes of polycation molecules and inorganic ions adsorbed on their surface. These differences become much more prominent with an increase in the size of the polycation and, accordingly, the charge introduced to the surface.

## 4. Polymer Layer at the Membranes of Mixed Composition

An important feature of polycations adsorption is their ability to create clusters of a negatively charged lipid component in the membrane under the polymer layer [112,113]. When the number of charged groups on the membrane surface is small, large, charged macromolecules may form locally heterogeneous structures upon the adsorption. Moreover, if the charge of the adsorbed polymer greatly exceeds the number of charged lipids in the membrane, the transfer of these lipids from the inner monolayer of the liposome (so-called “flip-flop”) is possible for further compensation of the polymer charge [114]. Lipid clustering upon the adsorption of polycations and other related phenomena have been demonstrated by MD methods in several works, discussed below. This phenomenon has been studied in several systems and it turns out that it does not depend on a specific polycation or charged lipid type. For instance, MD simulations show an increase in charged palmitoyl-oleoyl-phosphoserine (POPS) lipid density in the vicinity of the PMAPTAC polycation at the surface [75]. As a result, the adsorption of polymers with 20 and 40 units increase the amount of the charged PS molecules in their vicinity from 20 to 75 mol.%. A similar result was demonstrated for the adsorption of PEI and polyvinyl alcohol (PVA) on membranes composed of a mixture of dioleoyl-phosphoethanolamine/dioleoyl-phosphoglycerol (DOPE/DOPG) lipids [40]. Lateral diffusion and mobility of charged lipids in these membranes decrease in the presence of a polycation layer, which facilitates their clustering, while the mobility of zwitterionic lipids remains unchanged. Lipid clustering was also observed in the Monte Carlo simulations of a model polymer on the membrane from the mixture of phosphatidylinositol 4,5-bisphosphate/phosphoserine/phosphocholine (PIP_2_/PS/PC) [86,115]. Numerical simulations show that charged lipid clusters include mainly PIP_2_ molecules (4*e* per lipid), and their aggregation becomes more prominent with an increase in polycation degree of ionization [86] or polymerization [115]. Single-charged lipids PS aggregate under polycations only upon the shortage of PIP_2_ molecules needed for neutralization of the polymer charge. The efficiency of the clustering of charged lipids decreases with increasing ionic strength of the solution [86,87].

The polymer covering of the surface affects the solution in the diffuse part of the electric double layer. It has been studied systematically, and in much detail, on the example of several polycations in the work [40]. The authors showed that the adsorption of polycations leads to the reorientation of water molecules and the displacement of inorganic counterions (Na^+^) adsorbed on the membrane into the aqueous solution. The orientation of water molecules was characterized by the average value of the angle between the water dipoles and the normal to the membrane surface. The adsorbed polymer changes this angle, and this effect increases with the charge of the polycation molecules. The minimal effect was observed for the polyallylamine (PAA) polymer (having a charge of +3*e*), and the maximal for PEI and PLL (charge +10*e* and +20*e*, respectively). The efficiency of displacement of inorganic cations increased with the charge of the polycation molecules. Membrane overcharging becomes possible even if the charge introduced by the polymer to the surface is lower in its absolute value than the total charges of the membrane surface upon the polymer. This paradox was explained by the authors as a consequence of the incomplete displacement of Na^+^ ions from the membrane surface, that is, the overcharging is the result of the combined action of the charges of the polycations and counterions.

Briefly, adsorption of polycations leads to the following changes in the membrane (schematically illustrated by Figure 3): (*i*) dipole potential *φ_d_* decreases and surface potential *φ_s_* increases; (*ii*) the hydrogen bond network is partially reorganized; (*iii*) charged lipids cluster under the polymer layer due to their partial immobilization and ordering in the lateral direction; (*iv*) the membrane surface is overcharged and ζ-potential changes its sign; (*v*) water molecules reorient and counterions partially dislocate from the membrane surface to the diffuse part of the electric double layer above the polymer molecules. The profoundness of these effects is determined by the magnitudes of polycation and membrane charges, which depend on the degree of polymerization *N* and ionization *f* of macromolecules, as well as the ionic strength *I* of the solution. The larger the values of *N* and *f*, and the smaller the *I*, the more pronounced these effects are. Below, we show that the same parameters affect the efficiency of polycation binding to the membrane. According to data available in the literature, the presence of anionic lipids in the membrane mainly affects the binding efficiency of the polycation and its ability to overcharge the surface, while the structural rearrangements in the membrane are determined by the polymer size, side chains, and state of ionization of its chain. Steric limitations caused by the presence of large chains explain the relatively small effect of PLL on membrane properties, despite the significant charge of the polymer [40].

## 5. Conformations of Linear Polycation at the Charged Surface

Generally, upon adsorption to the surface, large macromolecules undergo a conformational transition from a three-dimensional state (random-coil-like) to a two-dimensional state (so-called “train-loop-tail conformation”) [42,87,116]. The state of train of some polymer’s regions assumes the direct contact of its bases with the surface. The polymer may have the region of loops, which consists of bases with no contacts with the surface besides two ends of the loop; and finally, a part of the polymer chain may be free as a tail exposed to the solution. The number and distribution of these regions along the polymer chain determine its conformation in the adsorption layer and depend mainly on the degree of its ionization and the charge density of the surface.

**Figure 4:**
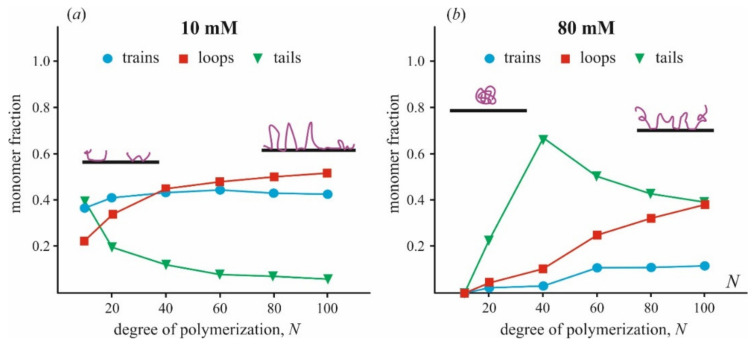
The relative fraction of polymer chain presented in train, loop, and tail conformations varied with a total number of monomer subunits *N* was calculated from data of [87] for two values of ionic strength (**a**) 10 mM, (**b**) 80 mM. Different states of polymer conformation are shown schematically. The fraction of unbound monomers is not presented in the plots.

A fraction of train, loop, and tail conformations in a polymer chain depends on the size of the polymer, *N*, and ionic strength, as is shown in Figure 4. These data are generalized by analysis of Monte Carlo simulations presented in the original publication [87]. One can see that when polymers at low ionic strength are tightly bound to the surface, the train fraction is about 40% and varies a little with the polymer length. The fraction of loops slowly increases with increasing polymer length, from 20% at *N* = 10 to 50% at *N* = 100. At high ionic strength, qualitatively different behavior of polymers is observed. A chain of polymer is bonded to the surface much weaker, and the fraction of trains is <10% for relatively long polymers (*N* > 50) and becomes negligible for short polymers. Most of the bases are exposed to the solution and presented in tails and loops. Similar results were obtained by the same method in [42], where the adsorption of a polyelectrolyte on small spherical particles was studied. The most noticeable difference in the conformation of adsorbed polycation molecules of different sizes is manifested only at low ionic strength. In this case, electrostatic interactions between polymer molecules and the surface, as well as between neighboring polymer molecules, play a significant role. This conclusion agrees with the results of a series of electrokinetic experiments carried out for several polymers (poly(tetramethyl-piperidenyloxyl-methacrylate) (PTMA) and poly(trimethylene carbonate) (PTMC)) adsorbed on the surface of latex particles [117,118,119]. The authors showed that the adsorption layer becomes much thinner in time at the excess of large polymer molecules in solution and low ionic strength. At high ionic strength and low molecular weight of polycation molecules, equilibrium is reached faster and leads to a homogeneous and thin adsorption layer.

The data presented in the above-cited works show the different states of polycation molecules at the surface. To analyze and describe these states, many authors developed theoretical models based on scaling theory [35,37,60,109]. This approach makes it possible to estimate the thickness of the adsorption layer *h* at different degrees of polymerization *N*, the degree of ionization *f*, and the surface charge density *σ*, as well as the ionic strength *I*. Some authors specify different surface charge densities that may determine different modes of the adsorption layer [37,59,60]. As it is shown in [60], at a charge density less than a certain value $\sigma <\sigma_{WC}=el_{B}^{-2}{\left(fN\right)}^{-3}$, the classical Poisson–Boltzmann approach works well for the description of the polymer near the charged surface (*l_B_* is the Bjerrum length). At densities of $\sigma >\sigma_{WC}$, the distance between charged macromolecules exceeds their average distance from the surface. In this mode, a strong repulsion between the chains organizes them on the charged surface in a strongly correlated Wigner liquid [120,121]. The equilibrium thickness is given by the expression $h\approx e/\left(l_{B}fN\sigma \right)$. At the density of $\sigma_{def}=e/\left(al_{B}f\cdot N^{3/2}\right)$, this thickness becomes comparable to the statistic two-dimensional size of the polycations $aN^{1/2}$, and the adsorption layer becomes more compact. With a further increase in *σ*, the layer thickness is determined by the balance of the electrostatic attraction energy and the confinement entropy of the polymer, which decreases due to the localization of its polymer chains at the surface. In this case, the layer thickness *h* does not depend on the degree of polymerization [37,60]:

(1) $$h=h_{0}={\left(\frac{a^{2}e}{l_{B}f\sigma }\right)}^{1/3}$$

The thickness of the adsorbed polycation layer of any degree of polymerization *N* and with the typical parameters of the system (*f* ~ 0.5, *a* ~ 0.3 nm, *l_B_* ~ 0.8 nm, *σ* ~ 0.7 *e*/nm^2^) is approximately equal to *h*_0_ = 0.7 nm. *σ* is the charge density defined as 0.5 *e* related to the characteristic cross-section area of lipid molecule equal to 70 Å^2^. A further increase in the surface charge density leads to the next change in the adsorption mode at $\sigma =\sigma_{e}=ef/a^{2}$; however, these values are experimentally and biologically irrelevant for lipid membrane systems. For example, for typical polycations *f* ~ 0.5 and *a* ~ 0.3 nm, the value of *σ_e_* ~ 18 *e*/nm^2^ is an order of magnitude higher than that of a fully charged lipid membrane (~1 *e*/nm^2^). As a result, this approach makes several verifiable predictions about the thickness of the adsorption layer. However, experimental data show a more complex and heterogeneous structure of the adsorption layer [84,97]. For this reason, a scaling theory approach should be expanded to take into account the structural features of the adsorption layer.

## 6. Lateral Heterogeneity of Polymer Layer as It Follows from the Analysis of Electrokinetic Data

Inhomogeneous features of the polymer layer are most convenient to consider on a membrane of mixed composition with different fraction *α* of the charged lipids. In this case, the structure of the polymer layer may be characterized by two parameters that can be controlled experimentally. They are a maximum fraction of the surface, *θ*_max_, occupied by the polycation, and the average thickness, *h*, of the polymer layer. Both parameters are related to the saturation state corresponding to the plateau discovered by *ζ*-potential measurements at an excessive concentration of polycation in the solution. In this respect, the most demonstrative are two cases: a neutral and fully charged membrane. At small fraction of a charged lipid, polycation molecules do not occupy the entire surface of the membrane and cover some parts of neutral lipids. This effect can be interpreted as the “spreading” of polycation molecules over the membrane surface. Similar structures of the adsorption layer were observed on the charged lipid in the membrane with varying of the fraction *α* [97]. In this work, the AFM method was used to study the structures formed by polylysines of different lengths induced by their adsorption on the surface of membranes with different fractions of anionic (cardiolipin, CL) and zwitterionic phospholipids (PC). Direct measurements showed that at *α* = 0.2, the value of *θ*_max_ increases from 0.3 to 0.45 with an increase in the degree of polymerization *N* from 5 to ~1000. This effect is more pronounced for molecules with a higher degree of polymerization. As was expected, the increased degree of polymerization leads to a thickened polymer layer from 1.5 nm for *N* = 5 to 2.5 nm for *N* ≈ 1000. The values of *θ*_max_ and *h* are in good agreement with the data of the MD simulations of similar systems. Thus, in [64], it was found *θ*_max_ = 0.12 for Lys_8_ and *θ*_max_ = 0.27 for Arg_8_ for a membrane made from a dimyristoyl-phosphocholine/dimyristoyl-phosphoglycerol (DMPC/DMPG) = 9:1 mixture (*α* = 0.1).

In membranes composed of only charged lipids, their local redistribution is not essential for polymer adsorption. They have no heterogeneous regions that may affect the structure of the adsorption layer. These membranes (*α* ~ 1) can be modeled as a surface with fixed charges. This assumption opens the possibility to consider a large number of works devoted to the adsorption of polycations on mica and latex particles: e.g., original studies in [122,123,124,125], and a review [126]. In the context of our review, the most interesting data is related to incomplete coverage of the surface by polylysines of a high degree of polymerization (*N* > 100) at a low ionic strength of solution (*I* ~ 10^−2^ M). In these series of experiments, the maximum surface coverage with the polymer *θ*_max_ varied from 0.5 to 0.6. Thus, according to [122,123], the saturation in the *ζ*-potential measurements occurred at *θ*_max_ ≈ 0.5. Our experiments show nearly the same values for adsorption of poly-L-lysine on mica measured by AFM [84]: *θ*_max_ ≈ 0.6 for *N* = 130 and *N* = 1000. These estimates fit into the concept of the random sequence adsorption (RSA) model. According to this concept, adsorbed molecules of large polyelectrolytes are considered as round disks irreversibly bound to the surface without the possibility of overlapping [126]. In this case, the fraction of the surface occupied by the polycation has an upper value, named the “jamming limit”, equal to *θ*_jam_ = 0.56. It means that even for *α* ~ 1, the maximal fraction of the surface *θ*_max_ occupied by large polycations should also be significantly less than 1. The assumption of the fixed shape (e.g., round) of adsorbed molecules can be attributed only to large polymers with *N* ~ 100 and higher, while for molecules with a low degree of polymerization, this is apparently not true. According to electrokinetic data, these molecules are not subjected to jamming, and may almost completely occupy the accessible surface, thus, for pentalysine, it produces *θ*_max_ = 0.9 [84]. The recorded thickness of the adsorption layer also varies with the value of *N*, from 1.5 nm for *N* > 100 to 0.8 nm for *N* = 5.

The formation of heterogeneous structures by polycation during adsorption is possible only under certain conditions. In addition to the high degree of polymerization, the appearance of the inhomogeneities is facilitated by the low ionic strength of the aqueous environment (~10^−2^ M). If it is increased, the electrostatic interaction between the surface and the adsorbed polycations is suppressed [118]. Numerical simulations carried out in [87,115] support this conclusion: the fraction of polymer chain exposed to the solution increased at high ionic strength. Moreover, when a certain concentration of background electrolyte was exceeded, polyelectrolyte molecules did not adsorb at the charged surface. The results of experiments on mica also demonstrated an increase in the thickness of the adsorption layer with an increase in ionic strength, suggesting conformational changes of polymer chains [127].

To investigate local inhomogeneities in the adsorption layer, we carried out a series of electrokinetic measurements of liposomes of varied compositions at low ionic strength (10^−2^ M) in the presence of synthetic polylysines of different lengths in the suspension [98,128]. To analyze the electrokinetic data, we developed a relatively compact analytical approach based on the simple Gouy–Chapman equation. It was combined with two analytical relations: a Langmuir-like adsorption isotherm to evaluate the polymer area coverage, and the Gouy–Chapman equation to determine the relationship between the surface charge density and *ζ*-potential. Charged and neutral lipids are assumed to be uniformly distributed in the membrane, in contrast to the polymer layer organized in clusters. The value of *ζ*-potential was calculated as surface-weighted mean considering different potentials above clusters and free membrane regions [129]. Surface charge density in cluster regions was calculated as a sum of lipid and polymer charges. For the sake of simplicity, we neglected the influence of the adsorbed polymer on the slipping plane position, and the charge distribution along the normal to the surface of the polymer layer was not taken into account. Three groups of polylysines were identified according to their degree of polymerization, *N*: short polymers, *N* ≤ 10; medium-length polymers, 10 < *N* ≤ 20; and long polymers, *N* > 20. These groups are qualitatively reflected in almost-coinciding experimental curves of the dependence of the *ζ*-potential on the amount of added polymer within each group. Quantitatively, these groups are characterized by values of the binding constants *K* with their values differing by almost an order of magnitude between groups: *K* > 0.5 × 10^4^ M^−1^ for short-length polymers; *K* ~ 4 × 10^4^ M^−1^ for medium, and *K* > 40 × 10^4^ M^−1^ for long ones [98]. The magnitude of these constants means that large polycations with high *K* value bind to the surface almost irreversibly, while short polymers remain in dynamic equilibrium with the solution.

We took into account the inhomogeneity of the adsorption layer on the membrane surface in the form of thickness *h* and occupied surface area *θ*_max_ dependence on *α*. We also carried out an MD simulation of experiments with pentalysine and polylysine of medium length (20 units). It was shown that only the assumption of the existence of inhomogeneity leads to results that agree with the MD data. Particularly, a raised number of polymer fractions exposed to the solution found in the MD simulation can be interpreted as an increased average thickness of the polymer layer. This result is in agreement with the data of numerical simulations carried out by other groups [64,87]. A lower number of polymer subunits *N* corresponds to its spreading on the surface and a uniform layer with a minimal thickness *h*_0_, which agrees with predictions of scaling theory [37,60]. The variation of the fraction *θ*_max_ with the parameters *α* and *N* is also consistent with the previously reported data [123,126]. In particular, *θ*_max_ for large polymers at *α* = 1 equals 0.6, as suggested by the theoretical RSA model.

**Figure 5:**
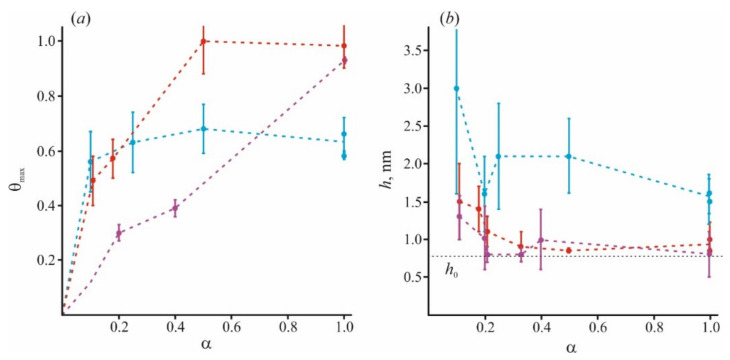
The fraction of the surface occupied by polycations of different sizes at the surface saturation state *θ_max_* (**a**) and the thickness of polymer layer *h* (**b**) are presented for the varied content of negatively charged lipid component. Data for the polymer of different groups are highlighted in color: blue for long polymers with *N* > 20, red for medium with 10 < *N* < 20, and magenta for short ones with *N* < 10. The dotted line denotes the minimal thickness of the adsorption layer *h*_0_ = 0.8 nm, according to AFM data for pentalysine [84].

**Figure 6:**
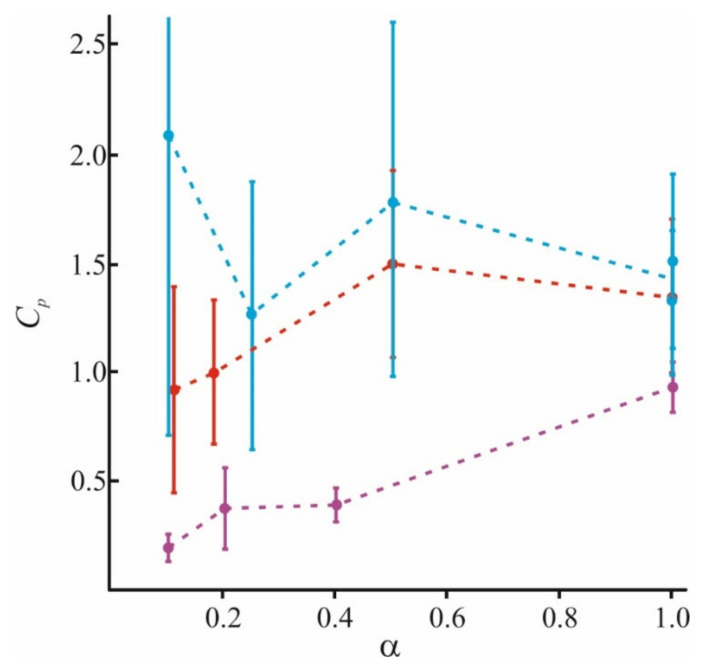
Calculated values of the membrane capacity *C_p_* to long (blue), medium (red), and short (magenta) polymers at a different fraction of charged lipids in the membrane *α*.

In Figure 5, we combine the literature data with those obtained by our group. In addition to our recent data of [98], we used results for small (Lys_5_) and large (Lys_130_) polymers at *α* = 1 and *α* = 0.2, and small (Lys_5_) polymers at *α* = 0.4 from [84,97], and for small (Lys_8_) polymers at *α* = 0.1 from [64]. The adsorption becomes more nonuniform for long-polymer molecules and at a small value of *α*. At any *α*, these molecules are unable to occupy the entire membrane surface (*θ*_max_ < 1) and cannot form a uniform layer without loops and tails (*h* > *h*_0_). At the same time, small molecules of Lys_5_ and Lys_8_ (magenta curves) occupy the area corresponding to the fraction of charged lipids in the mixture, spreading uniformly on the membrane surface as a thin layer. Polymers of medium length (red curves) behave intermediately: at small values of *α*, they form laterally inhomogeneous structures, since *θ*_max_ < 1, while at large *α*, they occupy almost the entire membrane. Parameters of our model allow to calculate the ratio of the total charge of the polymer layer to the total membrane charge at the saturation when the new portion of polymer molecules added to the water solution cannot bind to the surface. For simplicity, we will refer to this parameter as a capacity of the membrane to specific polymer, *C_p_*. This parameter is expressed as *C_p_* = *θ*_max_·*h*/*h*_0_ [98]. It reflects the adsorption efficiency of each type of polymer, and its ability to overcharge the surface. The values of *C_p_* at different *α*, presented in Figure 6, were calculated for polymers of different sizes.

The capacity of the membrane to polymers presented here is in good agreement with the data of [130] for adsorption of poly(N-methyl-2-vinylpyridinium methyl sulfate) (PVPQ) polymers at *α* = 0.18. According to these data, for long polymers, *C_p_* ≈ 2, and for medium, *C_p_* ≈ 1. The found value of *C_p_* is almost independent of *α* for long and medium polymers, as can be seen from Figure 6. At the same time, the adsorption layer properties substantially depend on *α*; the polymer layer is thick and inhomogeneous at low *α*, and thin and homogeneous at high *α*. As was expected, the adsorption efficiency grows with *N*. The capacity for pentalysine differs a little from unity even for the membranes completely filled with a charged lipid. This means that pentalysine molecules uniformly occupy almost the entire accessible surface, while large polymers form thick domains on the surface. The results obtained in [131] also indicate a decrease in membrane capacity *C_p_* with an increase in ionic strength *I* for medium and large polymers. A similar effect of ionic strength on the adsorption efficiency follows from experimental data [83] and numerical simulation results [87].

## 7. Concluding Remarks and Perspectives

The parameter *C_p_* supplies important information about the efficiency of polymer adsorption under various experimental conditions along with other characteristics of the polymer–membrane system. These are the thickness of the polymer layer *h* and the value of *θ*_max_, which qualitatively reflects the degree of its heterogeneity. Our current research focuses mainly on the electrokinetic data where these parameters cannot be measured directly. The typical adsorption isotherm corresponds to the amount of adsorbed substances or the fraction of its surface coverage to their concentration in the bulk of solution [132]. It describes adsorption by characteristics averaged over a surface, while fine details of polymer lateral distribution are of great importance, especially in the case of charged macromolecules. In our works, we prefer to analyze isotherms in terms of average surface coverage, since the fraction of the surface *θ*_max_ available for polymer adsorption determines the probability of their binding to the surface. However, this approach is possible only if the adsorption isotherm accounts for the coating thickness, which can significantly depend on the conditions and kinetics of polymer adsorption.

**Figure 7:**
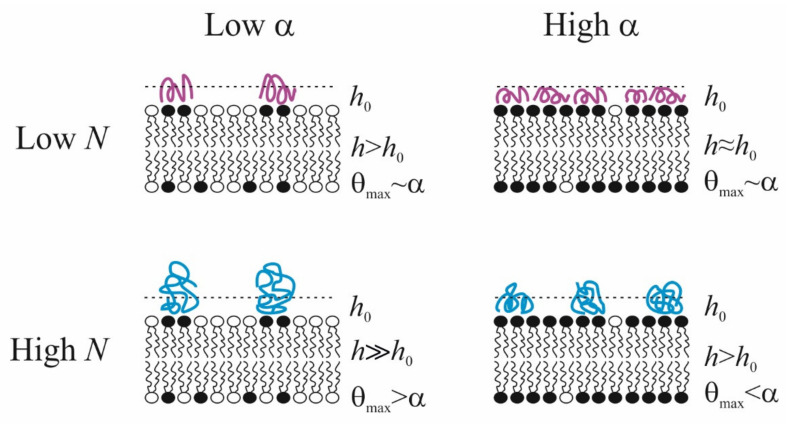
Schematic representation of the conformations of polycations with different degrees of polymerization *N* in an adsorption layer of thickness *h* on the surface of membranes with mixed composition *α*. *h*_0_ reflects the minimum polymer thickness in the train conformation.

Summarizing the above-presented information, we can formulate some conclusions about polycation adsorption at the membrane surface. (*i*) There is a discrepancy between the fraction of negatively charged lipids in their mix with the uncharged zwitterionic components in the membrane surface, *α*, and the fraction of the membrane area occupied by the adsorbed polycation (*θ*_max_). Namely, at small values of *α*, maximal area covered by polycations *θ*_max_ > *α*, and for large value of *α*, on the contrary, *θ*_max_ < *α*. The effect is more pronounced for polymers with a long chain: in particular, these macromolecules do not completely cover a fully charged membrane. (*ii*) The thickness of adsorption layer *h* increases with polymer length *N*. This rather obvious result is expressed in the number and size of tails and loops regions of the polymer exposed to the solution. (*iii*) If the fraction of charged lipid in the membrane *α* increases, then the thickness of the adsorption layer decreases, and polymer molecules spread out over the surface with a minimal value of the thickness *h*_0_, which corresponds to dimensions of subunits. Ultimately, both characteristics of the polymer layer allow reaching definite conclusions about its two possible states: homogeneous and inhomogeneous. The latter case is realized only at large *N* and at low ionic strength, especially at a small value of *α*, when a charge introduced by the polymer is much larger than charges located in the membrane. Thus, polymers, adsorbing on weakly charged lipid membranes, create clusters of negatively charged membrane component, and, to some extent, cover some neutral lipids also. On highly charged membranes, the steric repulsion between polymer molecules prevents them from covering the entire surface and leading to lateral inhomogeneity of the polymer layer. In the case of short polymers, the covering of the surface, *θ*_max_, is approximately equal to the fraction of charged lipids *α* in the membrane, while the thickness *h* shows the tendency to decrease up to its minimal value *h*_0_. The abovelisted conclusions are schematically illustrated in Figure 7.

We mentioned numerous experimental and theoretical works that convincingly demonstrate that electrostatic phenomena play the leading role in the interaction of polycations with a charged surface. This fact is considered by almost all theoretical works that describe the structure of the polymer layer and its relationship with the characteristics of charged macromolecules—their primary structure, chain length, presence of side groups, etc. As a rule, the theoretical approach to such systems implies the presence of negative charges fixed on the surface and the existence of a bulk positive charge in the adsorption layer. According to experimental data, the adsorption of polycation and multivalent ions fundamentally differ from each other. Under certain conditions, the adsorbed macromolecules do not occupy the entire available surface and can form polymer–lipid clusters, with each negative charge of the surface associated with several charges introduced by the polycation. An adequate description of this fact was quantitatively described by several theoretical approaches, including scaling theory, RSA, adsorption isotherms, and by direct solution of Poisson–Boltzmann equations. Theoretical models allow us to obtain the distribution of adsorbed substance over the surface in the lateral and normal directions, to predict the conformation of adsorbed macromolecules, and to perform a quantitative data analysis of electrokinetic measurements.

Solution of the Poisson–Boltzmann equations, as well as the derivation of the equations of the Gouy–Chapman model, accounts for the boundary conditions in which the surface charge density is fixed and assumed to be known, which is not always true for real objects. Fortunately, the model lipid membranes, such as liposomes, planar BLM, and lipid monolayers, allow us to control this important parameter with sufficient accuracy and even to vary it by mixing charged and neutral components. In our studies, we used this opportunity to assess important characteristics of the adsorption layer—its thickness and the degree of surface coverage. For this, we built an adsorption isotherm of polycations within the framework of a simplified model of the electric double layer [98,128,131]. As a first approximation, we assumed that charges of the polycation and lipid polar heads are located at the same plane at the liposome surface. Therefore, the calculation of the electric potential virtually ignores the adsorption layer dimensions, and the influence of the adsorbed polymer on the position of the slipping plane is also neglected. Nevertheless, the adsorption layer thickness defines the contribution of the polymer to the charge surface density of the liposome. Besides that, our model does not take into account the dependence of the polymer layer thickness on the membrane capacity. On the one hand, these assumptions simplified the derivation of the adsorption isotherm and allowed us to obtain a solution in the analytic form. On the other hand, they made analysis of the characteristics of the polymer layer beyond the saturation state impossible.

The development of experimental techniques greatly contributes to the evolution of theoretical models and the assessment of more realistic values of their parameters. In the context of this review, the electrostatic characteristics of the polymer–membrane system are of primary interest. Electrokinetic methods applied to different types of colloidal particles, such as liposomes, latex particles, and vesicles of biological origin, combined with registration of boundary potentials at planar BLM and Volta potentials of Langmuir lipid monolayers, allow us to obtain important information at the molecular level about structural reorganization in the area of polar heads groups induced by polymers. One example of this kind of data is shown above, in Figure 2. Assessment of the structural features of the polymer layer is significantly facilitated by direct measurements of surface structures on supported lipid bilayers by AFM methods as well as the analysis of the lateral distribution of adsorbent, available from the data of ellipsometry. Unfortunately, to date, such information is rarely accounted for in the majority of the physical models related to polymers at the surface of lipid and cell membranes. Our theoretical approach [98,128,131] contributes, to some extent, to the solution of this problem for electrokinetic data.

Despite the simplicity of the used theoretical approach, it provided a satisfactory description of the available experimental data. Nevertheless, in some conditions of electrokinetic measurements, i.e., at high ionic strength, this approach should be improved. While analyzing the inhomogeneity of polymer layer at the surface, we must take into account certain hydrodynamic effects of the system. For instance, the position of the slipping plane determining *ζ*-potential was considered in numerous works, in particular, by analytical models [133,134] and with a model of polymer chain by ball-like subunits [135]. Another important effect to be considered is distribution of polymer chain charges in the bulk of the adsorption layer together with its counterions [91,136]. At the same time, a significant role in the electrophoretic mobility of liposomes may be attributed to clusters of charged lipids under the adsorbed polymer. All mentioned corrections seem to be essential for relationships between the thickness of the adsorption layer and other characteristics of the system determined by the ionic strength of the solution, the degree of polymerization of the polycation, and the charge density on the membrane surface. Theoretical approaches developed by the scaling theory in [35,60] may be fruitful in this direction. This may reduce the number of independent parameters related to the inhomogeneity of the polymer layer and facilitate the approximation of experimental data. Finally, the development of the theoretical model will move it closer to practical application for a wider range of natural and synthetic macromolecules, biologically relevant polypeptides, and proteins with a complex structure composed from subunits of different states of ionization and hydrophobicity in their chains.

## Data Availability

No new data were created or analyzed in this study. Data sharing is not applicable to this article.

## References

[1] Biricova V., Laznickova A. (2009). Dendrimers: Analytical characterization and applications. Bioorg. Chem..

[2] Lee C.C., MacKay J.A., Fréchet J.M.J., Szoka F.C. (2005). Designing dendrimers for biological applications. Nat. Biotechnol..

[3] Timofeeva L., Kleshcheva N. (2011). Antimicrobial polymers: Mechanism of action, factors of activity, and applications. Appl. Microbiol. Biotechnol..

[4] Ai H., Jones S.A., Lvov Y.M. (2003). Biomedical Applications of Electrostatic Layer-by-Layer Nano-Assembly of Polymers, Enzymes, and Nanoparticles. Cell Biochem. Biophys..

[5] Svenson S., Tomalia D.A. (2012). Dendrimers in biomedical applications—Reflections on the field. Adv. Drug Deliv. Rev..

[6] Renault F., Sancey B., Badot P.-M., Crini G. (2009). Chitosan for coagulation/flocculation processes—An eco-friendly approach. Eur. Polym. J..

[7] Ikeda T., Ledwith A., Bamford C.H., Hann R.A. (1984). Interaction of a polymeric biguanide biocide with phospholipid membranes. Biochim. Biophys. Acta-Biomembr..

[8] Xue Y., Xiao H., Zhang Y. (2015). Antimicrobial Polymeric Materials with Quaternary Ammonium and Phosphonium Salts. Int. J. Mol. Sci..

[9] Carmona-Ribeiro A., de Melo Carrasco L. (2013). Cationic Antimicrobial Polymers and Their Assemblies. Int. J. Mol. Sci..

[10] Cho Y.W., Kim J.-D., Park K. (2003). Polycation gene delivery systems: Escape from endosomes to cytosol. J. Pharm. Pharmacol..

[11] Kabanov A.V. (1999). Taking polycation gene delivery systems from in vitro to in vivo. Pharm. Sci. Technol. Today.

[12] Park T., Jeong J., Kim S. (2006). Current status of polymeric gene delivery systems. Adv. Drug Deliv. Rev..

[13] Azevedo M.M., Ramalho P., Silva A.P., Teixeira-Santos R., Pina-Vaz C., Rodrigues A.G. (2014). Polyethyleneimine and polyethyleneimine-based nanoparticles: Novel bacterial and yeast biofilm inhibitors. J. Med. Microbiol..

[14] Beyth N., Houri-Haddad Y., Baraness-Hadar L., Yudovin-Farber I., Domb A.J., Weiss E.I. (2008). Surface antimicrobial activity and biocompatibility of incorporated polyethylenimine nanoparticles. Biomaterials.

[15] Illergård J., Römling U., Wågberg L., Ek M. (2012). Biointeractive antibacterial fibres using polyelectrolyte multilayer modification. Cellulose.

[16] Timofeeva L.M., Kleshcheva N.A., Shleeva M.O., Filatova M.P., Simonova Y.A., Ermakov Y.A., Kaprelyants A.S. (2015). Nonquaternary poly(diallylammonium) polymers with different amine structure and their biocidal effect on Mycobacterium tuberculosis and Mycobacterium smegmatis. Appl. Microbiol. Biotechnol..

[17] Baumgart T., Offenhäusser A. (2003). Polysaccharide-Supported Planar Bilayer Lipid Model Membranes. Langmuir.

[18] Diamanti E., Gregurec D., Rodríguez-Presa M.J., Gervasi C.A., Azzaroni O., Moya S.E. (2016). High Resistivity Lipid Bilayers Assembled on Polyelectrolyte Multilayer Cushions: An Impedance Study. Langmuir.

[19] Smith H.L., Jablin M.S., Vidyasagar A., Saiz J., Watkins E., Toomey R., Hurd A.J., Majewski J. (2009). Model Lipid Membranes on a Tunable Polymer Cushion. Phys. Rev. Lett..

[20] Van Meer G., Voelker D.R., Feigenson G.W. (2008). Membrane lipids: Where they are and how they behave. Nat. Rev. Mol. Cell Biol..

[21] Yaroslavov A., Efimova A., Lobyshev V., Kabanov V. (2002). Reversibility of structural rearrangements in the negative vesicular membrane upon electrostatic adsorption/desorption of the polycation. Biochim. Biophys. Acta-Biomembr..

[22] Antunes F.E., Marques E.F., Miguel M.G., Lindman B. (2009). Polymer–vesicle association. Adv. Colloid Interface Sci..

[23] Kahveci Z., Martínez-Tomé M., Esquembre R., Mallavia R., Mateo C. (2014). Selective Interaction of a Cationic Polyfluorene with Model Lipid Membranes: Anionic versus Zwitterionic Lipids. Materials.

[24] Ding L., Chi E.Y., Schanze K.S., Lopez G.P., Whitten D.G. (2010). Insight into the Mechanism of Antimicrobial Conjugated Polyelectrolytes: Lipid Headgroup Charge and Membrane Fluidity Effects. Langmuir.

[25] Kiss É., Heine E.T., Hill K., He Y.C., Keusgen N., Pénzes C.B., Schnöller D., Gyulai G., Mendrek A., Keul H. (2012). Membrane Affinity and Antibacterial Properties of Cationic Polyelectrolytes With Different Hydrophobicity. Macromol. Biosci..

[26] Kozon D., Mierzejewska J., Kobiela T., Grochowska A., Dudnyk K., Głogowska A., Sobiepanek A., Kuźmińska A., Ciach T., Augustynowicz-Kopeć E. (2019). Amphiphilic Polymethyloxazoline–Polyethyleneimine Copolymers: Interaction with Lipid Bilayer and Antibacterial Properties. Macromol. Biosci..

[27] Eisenberg M., Gresalfi T., Riccio T., McLaughlin S. (1979). Adsorption of Monovalent Cations to Bilayer Membranes Containing Negative Phospholipids. Biochemistry.

[28] Ermakov Y.A. (2000). Ion equilibrium near lipid membranes: Empirical analysis of the simplest model. Colloid J..

[29] Douglas J.F., Schneider H.M., Frantz P., Lipman R., Granick S. (1997). The origin and characterization of conformational heterogeneity in adsorbed polymer layers. J. Phys. Condens. Matter.

[30] Mbamala E.C., Ben-Shaul A., May S. (2005). Domain Formation Induced by the Adsorption of Charged Proteins on Mixed Lipid Membranes. Biophys. J..

[31] Tribet C., Vial F. (2008). Flexible macromolecules attached to lipid bilayers: Impact on fluidity, curvature, permeability and stability of the membranes. Soft Matter.

[32] Kim J., Mosior M., Chung L.A., Wu H., McLaughlin S. (1991). Binding of peptides with basic residues to membranes containing acidic phospholipids. Biophys. J..

[33] Samoshina Y., Nylander T., Shubin V., Bauer R., Eskilsson K. (2005). Equilibrium Aspects of Polycation Adsorption on Silica Surface: How the Adsorbed Layer Responds to Changes in Bulk Solution. Langmuir.

[34] Shubin V. (1994). Adsorption of Cationic Polymer onto Negatively Charged Surfaces in the Presence of Anionic Surfactant. Langmuir.

[35] Dobrynin A.V., Rubinstein M. (2005). Theory of polyelectrolytes in solutions and at surfaces. Prog. Polym. Sci..

[36] Tzlil S., Ben-Shaul A. (2005). Flexible Charged Macromolecules on Mixed Fluid Lipid Membranes: Theory and Monte Carlo Simulations. Biophys. J..

[37] Netz R.R., Andelman D. (2003). Neutral and charged polymers at interfaces. Phys. Rep..

[38] Gurtovenko A.A. (2019). Molecular-Level Insight into the Interactions of DNA/Polycation Complexes with Model Cell Membranes. J. Phys. Chem. B.

[39] Khomich D.A., Nesterenko A.M., Kostritskii A.Y., Kondinskaia D.A., Ermakov Y.A., Gurtovenko A.A. (2017). Independent adsorption of monovalent cations and cationic polymers at PE/PG lipid membranes. J. Phys. Conf. Ser..

[40] Kostritskii A.Y., Kondinskaia D.A., Nesterenko A.M., Gurtovenko A.A. (2016). Adsorption of Synthetic Cationic Polymers on Model Phospholipid Membranes: Insight from Atomic-Scale Molecular Dynamics Simulations. Langmuir.

[41] Tzlil S., Murray D., Ben-Shaul A. (2008). The “Electrostatic-Switch” Mechanism: Monte Carlo Study of MARCKS-Membrane Interaction. Biophys. J..

[42] Chodanowski P., Stoll S. (2001). Polyelectrolyte Adsorption on Charged Particles in the Debye−Hückel Approximation. A Monte Carlo Approach. Macromolecules.

[43] Silva R.A., Urzúa M.D., Petri D.F.S., Dubin P.L. (2010). Protein Adsorption onto Polyelectrolyte Layers: Effects of Protein Hydrophobicity and Charge Anisotropy. Langmuir.

[44] Alvares D.S., dos Santos Cabrera M.P., Ruggiero Neto J. (2016). Strategies for exploring electrostatic and nonelectrostatic contributions to the interaction of helical antimicrobial peptides with model membranes. Adv. Biomembr. Lipid Self-Assem..

[45] Zhao H., Mattila J.P., Holopainen J.M., Kinnunen P.K.J. (2001). Comparison of the membrane association of two antimicrobial peptides, magainin 2 and indolicidin. Biophys. J..

[46] Matos P.M., Franquelim H.G., Castanho M.A.R.B., Santos N.C. (2010). Quantitative assessment of peptide–lipid interactions. Biochim. Biophys. Acta-Biomembr..

[47] Chieng Y.Y., Chen S.B. (2011). Complexation of cationic polyelectrolyte with anionic phospholipid vesicles: Concentration, molecular weight and salt effects. J. Colloid Interface Sci..

[48] Hong S., Leroueil P.R., Janus E.K., Peters J.L., Kober M.M., Islam M.T., Orr B.G., Baker J.R., Banaszak Holl M.M. (2006). Interaction of polycationic polymers with supported lipid bilayers and cells: Nanoscale hole formation and enhanced membrane permeability. Bioconjugate Chem..

[49] Nievergelt A.P., Erickson B.W., Hosseini N., Adams J.D., Fantner G.E. (2015). Studying biological membranes with extended range high-speed atomic force microscopy. Sci. Rep..

[50] Sybachin A.V., Tsarkova L.A., Yaroslavov A.A. (2010). Atomic force microscopy of supported lipid membranes and their complexes with polycations. Biochem. Suppl. Ser. A Membr. Cell Biol..

[51] Ruths J., Essler F., Decher G., Riegler H. (2000). Polyelectrolytes I: Polyanion/Polycation Multilayers at the Air/Monolayer/Water Interface as Elements for Quantitative Polymer Adsorption Studies and Preparation of Hetero-superlattices on Solid Surfaces. Langmuir.

[52] de Meijere K., Brezesinski G., Möhwald H. (1997). Polyelectrolyte Coupling to a Charged Lipid Monolayer. Macromolecules.

[53] Pavinatto F.J., Pavinatto A., Caseli L., dos Santos D.S., Nobre T.M., Zaniquelli M.E.D., Oliveira O.N. (2007). Interaction of Chitosan with Cell Membrane Models at the Air−Water Interface. Biomacromolecules.

[54] Freire J.M., Domingues M.M., Matos J., Melo M.N., Veiga A.S., Santos N.C., Castanho M.A.R.B. (2011). Using zeta-potential measurements to quantify peptide partition to lipid membranes. Eur. Biophys. J..

[55] Yaroslavov A.A., Yaroslavova E.G., Rakhnyanskaya A.A., Menger F.M., Kabanov V.A. (1999). Modulation of interaction of polycations with negative unilamellar lipid vesicles. Colloids Surf. B Biointerfaces.

[56] Kabanov V., Yaroslavov A. (2002). What happens to negatively charged lipid vesicles upon interacting with polycation species?. J. Control. Release.

[57] Lorenz C.D., Faraudo J., Travesset A. (2008). Hydrogen Bonding and Binding of Polybasic Residues with Negatively Charged Mixed Lipid Monolayers. Langmuir.

[58] Troiano J.M., McGeachy A.C., Olenick L.L., Fang D., Liang D., Hong J., Kuech T.R., Caudill E.R., Pedersen J.A., Cui Q. (2017). Quantifying the Electrostatics of Polycation–Lipid Bilayer Interactions. J. Am. Chem. Soc..

[59] Carrillo J.-M.Y., Dobrynin A.V. (2007). Molecular Dynamics Simulations of Polyelectrolyte Adsorption. Langmuir.

[60] Dobrynin A.V., Deshkovski A., Rubinstein M. (2001). Adsorption of Polyelectrolytes at Oppositely Charged Surfaces. Macromolecules.

[61] Dobrynin A.V., Rubinstein M., Joanny J.-F. (1997). Adsorption of a Polyampholyte Chain on a Charged Surface. Macromolecules.

[62] Lasic D.D. (1997). The Conformation of Polymers at Interfaces.

[63] May S., Harries D., Ben-Shaul A. (2000). Lipid demixing and protein-protein interactions in the adsorption of charged proteins on mixed membranes. Biophys. J..

[64] Mcgeachy A.C., Caudill E.R., Liang D., Cui Q., Pedersen J.A., Geiger F.M. (2018). Counting charges on membrane-bound peptides. Chem. Sci..

[65] Schwieger C., Blume A. (2007). Interaction of poly(l-lysines) with negatively charged membranes: An FT-IR and DSC study. Eur. Biophys. J..

[66] Quemeneur F., Rinaudo M., Maret G., Pépin-Donat B. (2010). Decoration of lipid vesicles by polyelectrolytes: Mechanism and structure. Soft Matter.

[67] Quemeneur F., Rinaudo M., Pépin-Donat B. (2008). Influence of Polyelectrolyte Chemical Structure on their Interaction with Lipid Membrane of Zwitterionic Liposomes. Biomacromolecules.

[68] Papahadjopoulos D. (1968). Surface properties of acidic phospholipids: Interaction of monolayers and hydrated liquid crystals with uni- and bi-valent metal ions. Biochim. Biophys. Acta-Biomembr..

[69] Tocanne J.-F., Teissié J. (1990). Ionization of phospholipids and phospholipid-supported interfacial lateral diffusion of protons in membrane model systems. Biochim. Biophys. Acta-Rev. Biomembr..

[70] Zimmermann R., Freudenberg U., Schweiß R., Küttner D., Werner C. (2010). Hydroxide and hydronium ion adsorption—A survey. Curr. Opin. Colloid Interface Sci..

[71] Zimmermann R., Küttner D., Renner L., Kaufmann M., Zitzmann J., Müller M., Werner C. (2009). Charging and structure of zwitterionic supported bilayer lipid membranes studied by streaming current measurements, fluorescence microscopy, and attenuated total reflection Fourier transform infrared spectroscopy. Biointerphases.

[72] Tian C.S., Shen Y.R. (2009). Structure and charging of hydrophobic material/water interfaces studied by phase-sensitive sum-frequency vibrational spectroscopy. Proc. Natl. Acad. Sci. USA.

[73] Hartvig R.A., van de Weert M., Østergaard J., Jorgensen L., Jensen H. (2011). Protein Adsorption at Charged Surfaces: The Role of Electrostatic Interactions and Interfacial Charge Regulation. Langmuir.

[74] Weichselbaum E., Österbauer M., Knyazev D.G., Batishchev O.V., Akimov S.A., Hai Nguyen T., Zhang C., Knör G., Agmon N., Carloni P. (2017). Origin of proton affinity to membrane/water interfaces. Sci. Rep..

[75] Kopec W., Żak A., Jamróz D., Nakahata R., Yusa S., Gapsys V., Kepczynski M. (2020). Polycation–Anionic Lipid Membrane Interactions. Langmuir.

[76] Dalchand N., Cui Q., Geiger F.M. (2020). Electrostatics, Hydrogen Bonding, and Molecular Structure at Polycation and Peptide:Lipid Membrane Interfaces. ACS Appl. Mater. Interfaces.

[77] Wilkosz N., Jamróz D., Kopeć W., Nakai K., Yusa S., Wytrwal-Sarna M., Bednar J., Nowakowska M., Kepczynski M. (2017). Effect of Polycation Structure on Interaction with Lipid Membranes. J. Phys. Chem. B.

[78] Carrier D., Pezolet M. (1986). Investigation of polylysine-dipalmitoylphosphatidylglycerol interactions in model membranes. Biochemistry.

[79] Fleming E., Maharaj N.P., Chen J.L., Nelson R.B., Elmore D.E. (2008). Effect of lipid composition on buforin II structure and membrane entry. Proteins Struct. Funct. Bioinform..

[80] Kwolek U., Jamróz D., Janiczek M., Nowakowska M., Wydro P., Kepczynski M. (2016). Interactions of Polyethylenimines with Zwitterionic and Anionic Lipid Membranes. Langmuir.

[81] Marukovich N.I.I., Nesterenko A.M.M., Ermakov Y.A.Y.A. (2015). Structural factors of lysine and polylysine interaction with lipid membranes. Biochem. Suppl. Ser. A Membr. Cell Biol..

[82] Ermakov Y.A., Averbakh A.Z., Arbuzova A., Sukharev S.I. (1998). Lipid and cell membranes in the presence of gadolinium and other ions with high affinity to lipids. 2. A dipole component of the boundary potential on membranes with different surface charge. Membr. Cell Biol..

[83] Finogenova O.A., Filinsky D.V., Ermakov Y.A. (2008). Electrostatic effects upon adsorption and desorption of polylysines on the surface of lipid membranes of different composition. Biochem. Suppl. Ser. A Membr. Cell Biol..

[84] Finogenova O.A., Batischev O.V., Indenbom A.V., Zolotarevsky V.I., Ermakov Y.A. (2009). Molecular distribution and charge of polylysine layers at the surface of lipid membranes and mica. Biochem. Suppl. Ser. A Membr. Cell Biol..

[85] Hunter R., Ottewill R.H., Rowell R.L. (1981). Zeta Potential in Colloid Science: Principles and Applications.

[86] Duan X., Zhang Y., Li L., Zhang R., Ding M., Huang Q., Xu W.-S., Shi T., An L. (2017). Effects of Concentration and Ionization Degree of Anchoring Cationic Polymers on the Lateral Heterogeneity of Anionic Lipid Monolayers. J. Phys. Chem. B.

[87] Duan X., Zhang R., Li Y., Shi T., An L., Huang Q. (2013). Monte Carlo study of polyelectrolyte adsorption on mixed lipid membrane. J. Phys. Chem. B.

[88] Cevc G., Marsh D., Bittar E. (1987). Phospholipid Bilayers: Physical Principles and Models.

[89] Ermakov Y.A. (2021). Boundary Potential and the Energy of Lipid Monolayer Compression at the Liquid Expanded State. Biochem. Suppl. Ser. A Membr. Cell Biol..

[90] Ermakov Y.A., Averbakh A.Z., Yusipovich A.I., Sukharev S. (2001). Dipole Potentials Indicate Restructuring of the Membrane Interface Induced by Gadolinium and Beryllium Ions. Biophys. J..

[91] Vorotyntsev M.A., Ermakov Y.A., Markin V.S., Rubashkin A.A. (1993). Distribution of the interfacial potential drop in a situation when ionic solution components enter a surface layer of finite thickness with fixed space charge. Russ. Electrochem..

[92] Ermakov Y.A., Kamaraju K., Sengupta K., Sukharev S. (2010). Gadolinium Ions Block Mechanosensitive Channels by Altering the Packing and Lateral Pressure of Anionic Lipids. Biophys. J..

[93] Hoernke M., Schwieger C., Kerth A., Blume A. (2012). Binding of cationic pentapeptides with modified side chain lengths to negatively charged lipid membranes: Complex interplay of electrostatic and hydrophobic interactions. Biochim. Biophys. Acta-Biomembr..

[94] Ermakov Y.A. (2017). Boundary potential of lipid bilayers: Methods, interpretations and biological applications. J. Phys. Conf. Ser..

[95] Ermakov Y.A., Nesterenko A.M. (2017). Boundary potential of lipid bilayers: Methods and interpretations. J. Phys. Conf. Ser..

[96] Ermakov Y.A., Sokolov V.S. (2003). Boundary Potentials of Bilayer Lipid Membranes: Methods and Interpretations.

[97] Marukovich N., McMurray M., Finogenova O., Nesterenko A., Batishchev O., Ermakov Y. (2013). Interaction of Polylysines with the Surface of Lipid Membranes. The Electrostatic and Structural Aspects. Adv. Planar Lipid.

[98] Molotkovsky R.J., Galimzyanov T.R., Khomich D.A., Nesterenko A.M., Ermakov Y.A. (2021). Inhomogeneity of polylysine adsorption layers on lipid membranes revealed by theoretical analysis of electrokinetic data and molecular dynamics simulations. Bioelectrochemistry.

[99] Ermakov Y.A., Kamaraju K., Dunina-Barkovskaya A., Vishnyakova K.S., Yegorov Y.E., Anishkin A., Sukharev S. (2017). High-Affinity Interactions of Beryllium(2+) with Phosphatidylserine Result in a Cross-Linking Effect Reducing Surface Recognition of the Lipid. Biochemistry.

[100] McLaughlin S., Mulrine N., Gresalfi T., Vaio G., McLaughlin A. (1981). Adsorption of divalent cations to bilayer membranes containing phosphatidylserine. J. Gen. Physiol..

[101] Decher G. (1997). Fuzzy Nanoassemblies: Toward Layered Polymeric Multicomposites. Science.

[102] Sukhorukov G.B., Donath E., Davis S., Lichtenfeld H., Caruso F., Popov V.I., Möhwald H. (1998). Stepwise polyelectrolyte assembly on particle surfaces: A novel approach to colloid design. Polym. Adv. Technol..

[103] Ferreira M., Cheung J.H., Rubner M.F. (1994). Molecular self-assembly of conjugated polyions: A new process for fabricating multilayer thin film heterostructures. Thin Solid Film..

[104] Borukhov I., Andelman D., Orland H. (1999). Effect of Polyelectrolyte Adsorption on Intercolloidal Forces. J. Phys. Chem. B.

[105] de Vos W.M., Lindhoud S. (2019). Overcharging and charge inversion: Finding the correct explanation(s). Adv. Colloid Interface Sci..

[106] Shin Y., Roberts J.E., Santore M.M. (2002). The Relationship between Polymer/Substrate Charge Density and Charge Overcompensation by Adsorbed Polyelectrolyte Layers. J. Colloid Interface Sci..

[107] Schlenoff J.B., Dubas S.T. (2001). Mechanism of Polyelectrolyte Multilayer Growth: Charge Overcompensation and Distribution. Macromolecules.

[108] Joanny J.F. (1999). Polyelectrolyte adsorption and charge inversion. Eur. Phys. J. B.

[109] Joanny J.-F., Castelnovo M., Netz R. (2000). Adsorption of charged polymers. J. Phys. Condens. Matter.

[110] Brockman H. (1994). Dipole potential of lipid membranes. Chem. Phys. Lipids.

[111] Ermakov Y.A., Asadchikov V.E., Volkov Y.O., Nuzhdin A.D., Roshchin B.S., Honkimaki V., Tikhonov A.M. (2019). Electrostatic and Structural Effects at the Adsorption of Polylysine on the Surface of the DMPS Monolayer. JETP Lett..

[112] Yaroslavov A.A., Efimova A.A., Lobyshev V.I., Ermakov Y.A., Kabanov V.A. (1996). Reversibility of structural rearrangements in lipid membranes induced by adsorption-desorption of a polycation. Biol. Membr..

[113] Heimburg T., Angerstein B., Marsh D. (1999). Binding of peripheral proteins to mixed lipid membranes: Effect of lipid demixing upon binding. Biophys. J..

[114] Yaroslavov A.A., Melik-Nubarov N.S., Menger F.M. (2006). Polymer-Induced Flip-Flop in Biomembranes. Acc. Chem. Res..

[115] Duan X., Li Y., Zhang R., Shi T., An L., Huang Q. (2013). Regulation of anionic lipids in binary membrane upon the adsorption of polyelectrolyte: A Monte Carlo simulation. AIP Adv..

[116] Breen C. (1999). The characterisation and use of polycation-exchanged bentonites. Appl. Clay Sci..

[117] Adachi Y., Matsumoto T. (1996). Dynamics of initial stage flocculation of polystyrene latex spheres with polyelectrolytes. Colloids Surf. A Physicochem. Eng. Asp..

[118] Aoki K., Adachi Y. (2006). Kinetics of polyelectrolyte adsorption onto polystyrene latex particle studied using electrophoresis: Effects of molecular weight and ionic strength. J. Colloid Interface Sci..

[119] Feng L., Stuart M.C., Adachi Y. (2015). Dynamics of polyelectrolyte adsorption and colloidal flocculation upon mixing studied using mono-dispersed polystyrene latex particles. Adv. Colloid Interface Sci..

[120] Perel V., Shklovskii B. (1999). Screening of a macroion by multivalent ions: A new boundary condition for the Poisson–Boltzmann equation and charge inversion. Phys. A Stat. Mech. Its Appl..

[121] Shklovskii B.I. (1999). Wigner Crystal Model of Counterion Induced Bundle Formation of Rodlike Polyelectrolytes. Phys. Rev. Lett..

[122] Morga M., Adamczyk Z., Gödrich S., Oćwieja M., Papastavrou G. (2015). Monolayers of poly-l-lysine on mica—Electrokinetic characteristics. J. Colloid Interface Sci..

[123] Morga M., Adamczyk Z. (2013). Monolayers of cationic polyelectrolytes on mica—Electrokinetic studies. J. Colloid Interface Sci..

[124] Morga M., Adamczyk Z., Kosior D., Kujda-Kruk M. (2019). Kinetics of Poly-L-lysine Adsorption on Mica and Stability of Formed Monolayers: Theoretical and Experimental Studies. Langmuir.

[125] Finessi M., Sinha P., Szilágyi I., Popa I., Maroni P., Borkovec M. (2011). Charge Reversal of Sulfate Latex Particles by Adsorbed Linear Poly(ethylene imine) Probed by Multiparticle Colloidal Probe Technique. J. Phys. Chem. B.

[126] Szilagyi I., Trefalt G., Tiraferri A., Maroni P., Borkovec M. (2014). Polyelectrolyte adsorption, interparticle forces, and colloidal aggregation. Soft Matter.

[127] Porus M., Maroni P., Borkovec M. (2012). Structure of Adsorbed Polyelectrolyte Monolayers Investigated by Combining Optical Reflectometry and Piezoelectric Techniques. Langmuir.

[128] Molotkovsky R.J., Galimzyanov T.R., Ermakov Y.A. (2019). Polypeptides on the Surface of Lipid Membranes. Theoretical Analysis of Electrokinetic Data. Colloid J..

[129] Anderson J.L. (1985). Effect of nonuniform zeta potential on particle movement in electric fields. J. Colloid Interface Sci..

[130] Ivashkov O.V., Sybachin A.V., Efimova A.A., Pergushov D.V., Orlov V.N., Schmalz H., Yaroslavov A.A. (2015). The Influence of the Chain Length of Polycations on their Complexation with Anionic Liposomes. ChemPhysChem.

[131] Molotkovsky R.J., Galimzyanov T.R., Ermakov Y.A. (2021). Influence of Ionic Strength on Adsorption of Polypeptides on Lipid Membranes: Theoretical Analysis. Biochem. Suppl. Ser. A Membr. Cell Biol..

[132] Markin V.S., Volkova-Gugeshashvili M.I., Volkov A.G. (2006). Adsorption at Liquid Interfaces: The Generalized Langmuir Isotherm and Interfacial Structure. J. Phys. Chem. B.

[133] Donath E., Kuzmin P., Krabi A., Voigt A. (1993). Electrokinetics of structured interfaces with polymer depletion—A theoretical study. Colloid Polym. Sci..

[134] Ohshima H. (1995). Electrophoretic mobility of soft particles. Colloids Surf. A Physicochem. Eng. Asp..

[135] Hill R.J. (2004). Hydrodynamics and electrokinetics of spherical liposomes with coatings of terminally anchored poly(ethylene glycol): Numerically exact electrokinetics with self-consistent mean-field polymer. Phys. Rev. E.

[136] Hill R.J., Saville D.A. (2005). ‘Exact’ solutions of the full electrokinetic model for soft spherical colloids: Electrophoretic mobility. Colloids Surf. A Physicochem. Eng. Asp..

